# Clinical characteristics of depression in late life

**DOI:** 10.1192/j.eurpsy.2025.1357

**Published:** 2025-08-26

**Authors:** V. Pochueva, T. Safarova

**Affiliations:** 1 FSBSI Mental Health Research Center, Moscow, Russian Federation

## Abstract

**Introduction:**

The relevance of studying late-life depression is determined by the increase in its prevalence, difficulties in diagnosis and therapy. Depression in old age aggravates the course of comorbid somatic pathology, leads to a deterioration in the quality of life of the elderly, the development of mental insolvency and social maladjustment. In addition, depression accelerates the aging process, accompanied by an increased risk of mortality and the development of dementia.

**Objectives:**

To study the clinical features of late-life depression in patients of a gerontopsychiatric hospital.

**Methods:**

Patients with depressive disorders who were treated in the conditions of the gerontopsychiatric hospital. The study used clinical, psychopathological and statistical methods. A total of 333 patients were examined: 87 men (26.1%) and 246 women (73.39%) aged 60 and older (average age 69.05±6.66). According to the ICD-10 classification, all patients were diagnosed with a depressive episode of various etiologies: in 20 patients (6.0%) - a single DE, in 86 patients- a depressive phase within the framework of the BAR(25.9%)and in most patients–227people– a depressive phase within the framework of DDR. According to the ICD-10criteria, depression corresponded to a moderate depressive episode in 246 patients (73.9%), a mild depressive episode in 64 (19.2%)and a severe depressive episode in 23 patients (6.9%).

**Results:**

Analysis of the psychopathological structure of the examined inpatient patients with late-age depression revealed the predominance of psychopathological conditions characteristic of late age, in the structure of which anxiety and anxiety-melancholy disorders dominated (152people-45.7% of cases), depression with the dominance of apathetic and adynamic disorders, which were observed in 135people (40.5%), were somewhat less common. Complex psychopathological depressions with a predominance of senesto-hypochondriac disorders, as well as delusional depressions, were less common – in 39 people–11.7% and 7 people-2.1% of cases, respectively. A characteristic feature of late-age depressions was the presence in their structure of dissomnic, anxiety and hypochondriac disorders, which were noted, respectively, in100%, 94.6% and 93.1% of cases, as well as subclinical cognitive disorders: subjective cognitive impairment (complaints of memory loss)- in 64.3% of patients.

**Conclusions:**

The study shows the polymorphism of psychopathological symptoms of endogenous depression in late life, the presence of anxiety, senesto-hypochondriac, dissomnic and cognitive disorders in the structure of depression.

**Disclosure of Interest:**

None Declared

